# Know Your Current *I_h_*: Interaction with a Shunting Current Explains the Puzzling Effects of Its Pharmacological or Pathological Modulations

**DOI:** 10.1371/journal.pone.0036867

**Published:** 2012-05-11

**Authors:** Michele Migliore, Rosanna Migliore

**Affiliations:** Institute of Biophysics, National Research Council, Palermo, Italy; Sackler Medical School, Tel Aviv University, Israel

## Abstract

The non-specific, hyperpolarization activated, *I_h_* current is particularly involved in epilepsy and it exhibits an excitatory or inhibitory action on synaptic integration in an apparently inconsistent way. It has been suggested that most of the inconsistencies could be reconciled invoking an indirect interaction with the *M*-type *K^+^* current, another current involved in epilepsy. However, here we show that the original experiments, and the simplified model used to explain and support them, cannot explain in a conclusive way the puzzling *I_h_* actions observed in different experimental preparations. Using a realistic model, we show instead how and why a shunting current, such as that carried by TASK-like channels, and dependent on *I_h_* channel is able to explain virtually all experimental findings on *I_h_* up- or down-regulation by modulators or pathological conditions. The model results suggest several experimentally testable predictions to characterize in more details this elusive and peculiar interaction, which may be of fundamental importance in the development of new treatments for all those pathological and cognitive dysfunctions caused, mediated, or affected by *I_h_*.

## Introduction

Experimental findings on the effects of *I_h_* modulation appear to be inconsistent. Although some results can be explained in terms of *I_h_* intrinsic properties and dendritic distribution [1], its real nature, effects, and possible interaction with other membrane mechanisms are poorly understood (discussed in [2]). The major source of confusion is that, from its reversal potential around −30 mV, it can be expected to exert an excitatory action. However, in many experiments on hippocampal neurons it exhibits a surprising inhibitory effect, making the underlying mechanism of action and its possible relevance for therapeutic applications far from clear. A striking example of the kind of problems faced in interpreting experimental findings can be found in CA1 pyramidal neurons: induction of febrile seizures [3] or application of the anticonvulsant agent lamotrigine [4] both cause an *I_h_* up-regulation, but result in opposite effects (excitation and inhibition with respect to control, respectively) during dendritic current injections. It has been recently suggested [5] that most of the inconsistencies among the experimental (and as well as modeling) findings related to *I_h_* could be explained by an indirect interaction with the *M*-type potassium current (*K_M_*). The inhibitory (instead of the expected excitatory) effect, observed during a synaptic stimulation, was thus interpreted with an increased activation of *K_M_* overcompensating the higher excitability generated by the more depolarized resting membrane potential (RMP) in the presence of *I_h_*. However, in CA1 pyramidal neurons the *K_M_* is localized in the axo-somatic region [6]–[7], and the rare channels found in the dendrites [8] do not seem to affect synaptic integration [9]. We thus reasoned that is unlikely for *K_M_* to play a significant role in modulating the effects of *I_h_*, which is instead predominantly involved with synaptic integration and with a predominant dendritic distribution. This is an extremely timely and intriguing issue, given the particularly important functional role that both *K_M_* and *I_h_* play in epilepsy [4], [10]–[11]. Unfortunately, as we discuss in this paper, recent experiments and the simplified models used to explain and support them, cannot explain in a conclusive way the puzzling *I_h_* actions observed in different experimental preparations. Here, using a realistic model we show instead how and why a shunting current, such as that carried by TASK-like channels [12]–[13], dependent on the *I_h_* peak conductance is able to explain virtually all experimental findings on *I_h_* up- or down-regulation by modulators or pathological conditions.

## Materials and Methods

**Figure 1:**
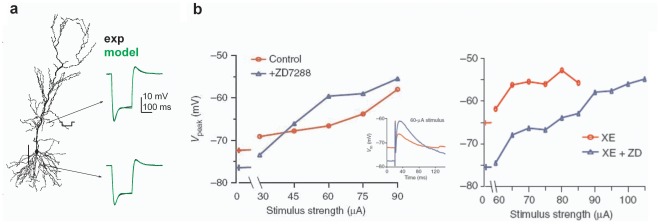
Realistic modeling of puzzling experimental findings. **a**) the 3D reconstruction used in most simulations (*left*, cell ri06 from the neuromorpho.org database), and model fitting (*green*) of simultaneous dendritic and somatic experimental recordings (*black*); dendritic current injection (1 nA, 200 ms, ∼200 µm from soma); cell’s scale bar is 100 µm; **b**) (*left*) Typical peak somatic depolarization reached during dendritic stimulations in an experiments with (*red*) or without (*blue*) *I_h_*; inset shows somatic recordings for a 60 µA stimulus; (*right*) Peak somatic depolarization with or without *I_h_* after block of *K_M_*. Experimental results in panel *b* report results observed in different CA1 neurons, and were adapted from Figs. 2b and 6c of [5] with permission from Macmillan Publishers Ltd, copyright (2009).

All simulations were implemented with the NEURON program [14], and model files are available for public download under the ModelDB section of the Senselab database (http://senselab.med.yale.edu). We started from a morphologically accurate model of a CA1 neuron with active and passive properties already validated against a number of different experimental findings [15], including sodium and delayed rectifier potassium conductances uniformly distributed throughout the dendrites, an *A*-type potassium [16] conductance linearly increasing with distance from the soma, a *K_M_* in the axosomatic region [7], [10], [17], and *I_h_* [4], [18]. The passive properties and *I_h_* parameters were optimized to simultaneously fit both the somatic and dendritic responses of a CA1 pyramidal neuron to a dendritic current injection under physiological conditions, as shown in Fig. 1a [15]. For the purposes of this paper, the *I_h_* current was modeled with an additional component, *I_lk_*:
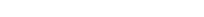
where *g_h_*(*x*) is the local peak conductance (at *x* µm from the soma), *l* is the activation variable (from [4]), and *v_rev_h_* = −30 mV. The *I_lk_* was implemented as *I_lk_ = g_h_*(*x*)·*lk*·(*v*–*v_rev_lk_*), with a voltage- and time-independent parameter, *lk,* and a reversal potential of *v_rev_lk_*. Unless explicitly noted otherwise, *lk* = 0. Note that changes or different values for *g_h_*(*x*) (e.g. because of a different dendritic location or ZD7288 application) would also affect this current, and that it will have a shunting effect for *v_rev_lk_* in the range of the resting membrane potential. It should also be stressed that we used this formulation as a convenient way to implement the explicit dependence of *I_lk_* from *I_h_* channels, and it does not imply any change in the conventional *I_h_* channel kinetic and activation properties. As pointed out later in the paper (see Discussion), although with our model we can make a few experimentally testable predictions, the detailed nature and properties of this current remain to be experimentally investigated.

All simulations were implemented with the NEURON program [14], and model files are available for public download under the ModelDB section of the Senselab database (http://senselab.med.yale.edu). We started from a morphologically accurate model of a CA1 neuron with active and passive properties already validated against a number of different experimental findings [15], including sodium and delayed rectifier potassium conductances uniformly distributed throughout the dendrites, an *A*-type potassium [16] conductance linearly increasing with distance from the soma, a *K_M_* in the axosomatic region [7], [10], [17], and *I_h_* [4], [18]. The passive properties and *I_h_* parameters were optimized to simultaneously fit both the somatic and dendritic responses of a CA1 pyramidal neuron to a dendritic current injection under physiological conditions, as shown in Fig. 1a [15]. For the purposes of this paper, the *I_h_* current was modeled with an additional component, *I_lk_*:
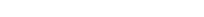
where *g_h_*(*x*) is the local peak conductance (at *x* µm from the soma), *l* is the activation variable (from [4]), and *v_rev_h_* = −30 mV. The *I_lk_* was implemented as *I_lk_ = g_h_*(*x*)·*lk*·(*v*–*v_rev_lk_*), with a voltage- and time-independent parameter, *lk,* and a reversal potential of *v_rev_lk_*. Unless explicitly noted otherwise, *lk* = 0. Note that changes or different values for *g_h_*(*x*) (e.g. because of a different dendritic location or ZD7288 application) would also affect this current, and that it will have a shunting effect for *v_rev_lk_* in the range of the resting membrane potential. It should also be stressed that we used this formulation as a convenient way to implement the explicit dependence of *I_lk_* from *I_h_* channels, and it does not imply any change in the conventional *I_h_* channel kinetic and activation properties. As pointed out later in the paper (see Discussion), although with our model we can make a few experimentally testable predictions, the detailed nature and properties of this current remain to be experimentally investigated.

To take into account the latest available experimental data [19], the peak *I_h_* conductance was modeled with a sigmoid increase with distance from the soma as:
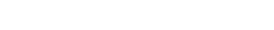
where *g_h_pk_* is the somatic peak density, *x* is the distance from soma (in µm), and the constants *x0* and *s* define the midpoint and shape of the sigmoid, respectively. The values of the fitted parameters obtained using the *Multiple Run Fitter* tool of NEURON are reported in Table 1.

**Table 1:** Parameter values best fitting the experimental traces in Fig. 1a.

g_h_ (mS/cm^2^)	*x_0_* (µm)	*s* (µm)	R_m_ (kΩ cm^2^)	C_m_ (µF/cm^2^)	R_a_ (Ω•cm)	*lk* (%)	*v_rev_lk_* (mV)	error (mV^2^)
0.007	340	30	20.0	1.9	80	3.7	−65.61	0.44

The *K_M_* current was added to the soma and axon, using the same model previously used to study its functional role in CA1 pyramidal neurons [7], [10], [17]. Currents at rest were not compensated to set the resting potential, and a reversal potential of −75 mV was used for the passive leakage mechanism. To model the somatic depolarization generated by a stimulating extracellular electrode in stratum radiatum (as in [5]), 50 excitatory synapses of up to 0.4 nS were modeled as a double exponential conductance change (with rise and decay time of 0.5 and 20 ms, respectively, and reversal potential of 0 mV) and randomly distributed in the oblique dendrites 100–500 µm from the soma. Test simulations using different random distributions gave the same qualitative results.

## Results

One of the controversial experimental findings that we will discuss here is the peak somatic depolarization reached during the activation of dendritic synaptic inputs on hippocampal CA1 pyramidal neurons, reported by George et al. [5]. The results for a specific cell, illustrated in Fig. 1b (left), show that the curve for the peak somatic depolarization under control conditions crossed that obtained without *I_h_* (i.e. with ZD7288). The crossover effect is important, because it demonstrates that the *I_h_* can enhance or inhibit the spike firing for weak or strong inputs, respectively, with possible consequences on the generation and spreading of seizures. The inhibitory effect is illustrated in the inset of Fig. 1b (left) for a particular stimulus strength. Next, in a different experiment (in different cells) carried out in the presence of the *K_M_* blocker XE991, the authors found that the peak somatic depolarization with or without *I_h_* never showed a crossover effect, with the *I_h_* exhibiting an excitatory action over the entire range of input strength tested (Fig. 1b, right, compare red and blue symbols). This result was interpreted as caused by the block of *K_M_*, and it was thus suggested that a *K_M_*−*I_h_* interaction could be responsible for the inhibitory effect of *I_h_* on EPSPs.

**Figure 2:**
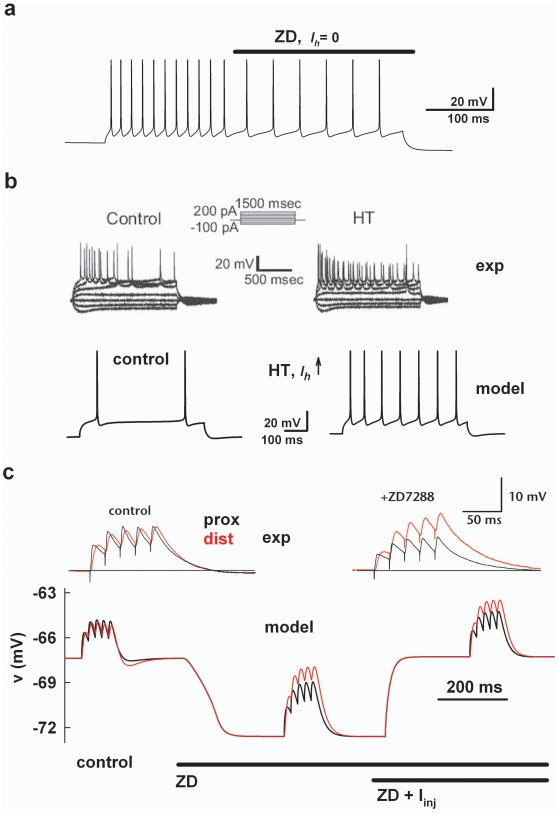
Typical experimental and model findings on *I_h_*. **a**) simulation of ZD7288 application during a 0.33 nA somatic current injection: at *t* = 500 the *I_h_* was blocked by resetting the peak conductance to 0; **b**) (*top*) increase in the dendritic firing rate after *I_h_* upregulation following febrile seizures induction (adapted from Fig. 2A of [3]); (*bottom*) simulation of *I_h_* upregulation in febrile seizures; traces are dendritic recordings during a 500 ms current injection (0.4 nA at ∼280 µm), using a 3× increase in *g_h_pk_* (from 0.01 mS/cm^2^) to obtain about the same depolarization (∼3 mV) and the same increase (∼3×) in the number of APs observed in the experiments; **c**) (*top*) experimental recordings demonstrating an increase in temporal summation at the soma during distal dendritic EPSPs after ZD2288 application (taken and redrawn from Fig. 1b of [22], with permission by Macmillan Publishers Ltd, copyright (1999)); simulation of EPSPs temporal summation during a 50 Hz train of 5 dendritic EPSPs activated under different conditions; the bars above the plots represent the timing of *I_h_* block (modeling ZD7288 application), and a somatic current injection (0.11 nA) modeling the experimental protocol to restore the original membrane resting potential after ZD7288 [22]; traces are somatic recordings during proximal (24 µm) or distal (500 µm) stimulation of the main trunk; peak synaptic conductances (1.7 and 5 nS for proximal and distal stimulations, respectively) were adjusted to obtain the same peak somatic depolarization during the first EPSP under control conditions; *g_h_pk_* = 0.01 mS/cm^2^.

To investigate in more details these results, we started by validating and testing the limits of our model simulating many different experimental findings on *I_h_* under different conditions. We began by modeling the effect of the *I_h_* blocker ZD7288 (ZD). During a 900 ms somatic current injection, the *I_h_* was blocked by resetting its peak conductance to 0 at t = 450 ms (Fig. 2, *ZD*). The result was a reduction of the spike frequency, and it is consistent to what observed in experimental studies using ZD (e.g. [20]–[21]). This effect can be interpreted as caused by the lower resting membrane potential (RMP) induced by the suppression of the excitatory driving force towards the *I_h_* reversal potential. We next modeled the *I_h_* up-regulation experimentally observed following febrile seizures as an overall 3× increase in the peak *I_h_* conductance. In agreement with experiments (Fig. 2b, top traces, from ref. [3]), this resulted in a higher RMP (∼3 mV) and an increase (∼3×) in the number of APs generated by the same dendritic stimulation (Fig. 2b, bottom traces). Finally, to show the effect of *I_h_* on synaptic integration, we modeled a classic experimental protocol activating a train of proximal or distal synaptic inputs (5 pulses at 50 Hz with and without *I_h_*). Typical experimental findings are shown in Fig. 1c (top traces, from ref. [22]). In the model, the proximal or distal train was activated during the same simulation under three different conditions: 1) control (Fig. 2c, bottom traces, *control*), 2) after ZD (Fig. 2c, bottom traces, *ZD*) and, 3) after a somatic current injection added to compensate for the hyperpolarization induced by ZD application (Fig. 2c, bottom traces, *ZD+I_inj_*). Again as in the experiments [22], the simulation showed that *I_h_* normalizes the temporal summation at the soma of a train of dendritic EPSPs (Fig. 2c, compare black and red traces under *control*). However, the overall excitatory or inhibitory effect (in terms of the peak depolarization reached during the train) depends on the specific experimental conditions (discussed in [2]) and, in particular, by the additional current injection routinely used in the experiments to compensate for the change in RMP after ZD (Fig. 2c, compare traces under *ZD* and *ZD+I_inj_*). These results demonstrate that a “naive” *I_h_*, without any direct or indirect association with another mechanism, is able to take into account different experimental findings.

**Figure 3:**
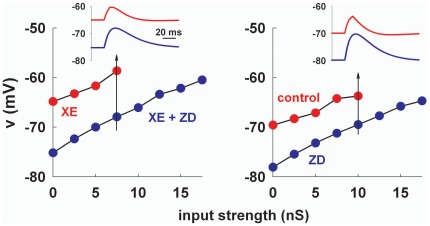
A dynamic interaction between *I_h_* and *K_M_* in a realistic model cannot reproduce the experimental findings. Peak somatic membrane potential as a function of synaptic input strength, without (*left*) or with (*right*) *K_M_*, and with (*red*) or without (*blue*) *I_h_*. Insets show somatic traces for a 7.5 nS (*left*) or a 10 nS (*right*) synaptic input.

We next considered the peak somatic depolarization reached during the activation of dendritic synaptic inputs on hippocampal CA1 pyramidal neurons (Fig. 1b). In order to reproduce the control conditions of the experiments discussed in George et al [5], we first adjusted the peak *I_h_* conductance in such a way to match the RMP and peak depolarization obtained in the experiments using the *K_M_* blocker XE991, with and without *I_h_* (Fig. 3, left, compare with right panel in Fig. 1b). As in the experiments, the peak somatic depolarization was higher in the presence of *I_h_* (red symbols in Fig. 3) over the entire range of input strength tested. This excitatory effect of *I_h_* was interpreted as caused by the block of *K_M_*. In other words, under control conditions, the *K_M_* should be strong enough to reduce the peak depolarization in such a way to generate a crossover effect between the curves obtained with or without *I_h_*. However, our simulation for this case did not show any crossover effect (Fig. 3, right). The results suggested that a *K_M_* would indeed reduce the peak depolarization. However, this would occur with or without *I_h_*, preventing in most cases a crossover effect. These results thus show a possible problem in interpreting the experimental findings, and in this work we argue that, under physiological conditions, the suggested interaction between *K_M_* and *I_h_* cannot explain the observed behavior unless some other (so far missed) mechanism is taken into account.

**Figure 4:**
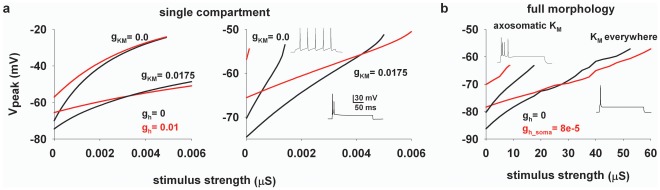
A dynamic interaction between *I_h_* and *K_M_* can show a crossover effect only in special cases. **a**) (*left*) peak somatic membrane potential as a function of stimulus strength in a non spiking single-compartmental model, with (*red* traces) or without *I_h_* (*black* traces) and different values for the *K_M_* peak conductance, *g_KM_*; note the large depolarization with *g_KM_* = 0; (*right*) same as in the *left* panel but using a spiking single-compartmental model; insets show somatic potential during a current clamp of 0.5 or 0.05 nA with or without *K_M_*, respectively. **b**) peak somatic membrane potential as a function of stimulus strength using the full realistic morphology with (*red* traces) or without *I_h_* (*black* traces) and two different *K_M_* channel distributions; insets show somatic potential during a current clamp in the two cases, respectively.

**Figure 5:**
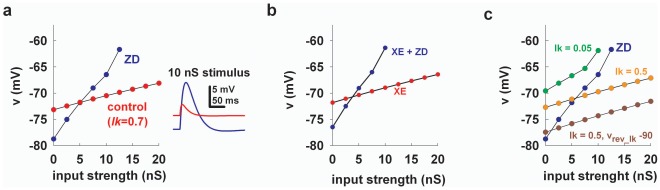
A shunting current proportional to *I_h_* takes into account all experimental findings. **a**) (*left*) peak somatic membrane potential as a function of synaptic stimulation strength using *I_lk_* with *lk* = 0.7, i.e. 70% of the peak *I_h_* conductance) under control (*red*) and no *I_h_* (*blue*); inset shows somatic recordings during a 10 nS stimulus; *g_KM = _*10 ms/cm^2^; **b**) same as in panel *a* but without *K_M_*; **c**) peak somatic membrane potential as a function of synaptic input strength without *I_h_* (*blue*), with *I_h_* and different values of *lk* (*green* and *orange*), or with *v_rev_lk_* = −90 mV.

**Figure 6:**
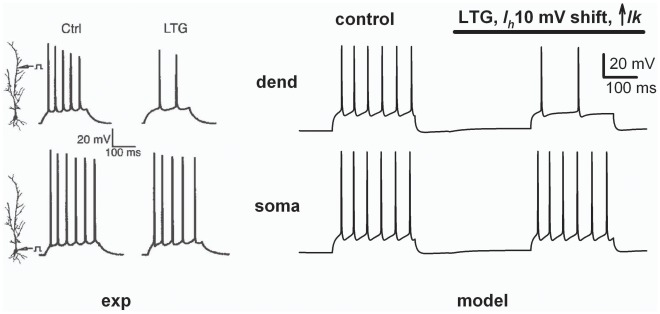
Modeling experimental findings on lamotrigine application. (*left*) experimental recordings demonstrating a decrease in dendritic (but not somatic) firing after *I_h_* up-regulation caused by application of the anticonvulsive drug Lamotrigine (adapted from Fig. 2d of [4]); (*right*) simulation of somatic (*bottom*) and dendritic (*top*, ∼200 µm from soma) recordings during a 500 ms current injection of 0.55 or 0.82 nA, respectively, before (control) and during LTG application (bar above plots); *g_h_pk_* = 0.01 mS/cm^2^, *lk* = 0.5 (control), *lk* = 0.8 (LTG).

Considering the *K_M_* channel’s properties the results discussed above are not surprising. It should be easy to realize that any modulation increasing the total *K_M_* current, in the attempt to obtain a crossover point (e.g. a different activation curve or dendritic distribution), would just increase the membrane hyperpolarization towards the *K^+^* reversal potential. It is important to stress that this effect will occur in all cases, including any pharmacological manipulations that do not affect the *K_M_*, such as a ZD application. Only under very special circumstances the effect of *K_M_* might result in a range of synaptic strength for which *I_h_* would have an inhibitory effect. A particular example is an electrotonically very compact neuron that can be represented as a single-compartment model, as in [5]. In order to better clarify this issue, we reproduced all the modeling results by George et al. [5]. The main result in those cases was obtained using a non-spiking (i.e. without *Na* ^+^ channels) soma-only configuration, with a crossover point modulated by the *K_M_* and *I_h_* peak conductance, as shown in Fig. 4a (*left*). However, using a spiking soma (Fig. 4a, *right*, not tested in [5]) reveals that the amount of *K_M_* conductance needed to obtain a crossover point consistent with experiments is quite unrealistic, since it should be so strong to block any repetitive spiking activity (Fig. 4a, *right*, insets), in striking contrast with any experimental evidence on CA1 neurons. Even worse were the results using the multi-compartmental, but still electrotonically very compact, morphology used in [5] (not shown), or our full CA1 morphology using different *K_M_* distributions (Fig. 4b). In all cases, the *K_M_* needed to obtain a reasonable crossover effect was too high to allow repetitive firing at any input current (Fig. 4b, insets show typical cases). Thus, it is unlikely that this current can play a main role. The reason for this failure is that a dendritic synaptic input reaches a suprathreshold value before any significant inhibitory *I_h_* effect can be observed at the soma. These results demonstrate that a simple interaction between *K_M_* and *I_h_* alone cannot explain the experimental findings, and suggest that some additional mechanism is missing.

Using an additional current proportional to the *I_h_* peak conductance, *I_lk_*, we were able to model the excitatory and inhibitory effect of *I_h_* in very good agreement with the experiments, as shown in Fig. 5a: *I_h_* increases the peak depolarization for weak inputs and reduces it for stronger inputs (a typical case of somatic potential for a strong input is shown in the inset). Blocking the *K_M_* did not change much the result (Fig. 5b). Instead, using different values of *lk* and *I_lk_* reversal potential we were able to generate a crossover point between excitation and inhibition practically anywhere over the entire range of synaptic strength (Fig. 5c), just as observed in the experiments [5]. These results thus suggest that the puzzling excitatory/inhibitory effect of *I_h_* can be explained in terms of a shunting current dependent on *I_h_* channels.

We finally tested our model against one of the clearest experimental findings on the different effect at the soma and dendrites caused by *I_h_* upregulation, i.e. during application of the anticonvulsant drug lamotrigine [4]. In the experiments, lamotrigine caused +10 mV shift of *I_h_* activation and a consequent ∼3 mV depolarization of the resting membrane potential. These changes should produce an overall increase in cell’s excitability. However, they instead resulted in a negligible effect at the soma and a strong inhibitory action in the dendrites, as shown in Fig. 6 (*left*). With our model, these results can be straightforwardly explained by modeling LTG application with a 60% increase of *lk*, as shown in Fig. 6 (*right*). Similar results were obtained using different dendritic *I_h_* distributions (not shown). It may be questioned that LTG may also affect *Na* ^+^ current, suggesting an alternative explanation for the reduced excitability after its application. This possibility, however, can be excluded by noting that in the experiments somatic traces were unaffected by LTG application (see somatic recordings in Fig. 6 left, from ref [4]). Taken together, these results further demonstrate that an additional current coupled to *I_h_* channels is able to take into account practically all experimental findings on the effects of *I_h_* under different conditions.

## Discussion

The kinetic, activation properties, and dendritic distribution of *I_h_* cannot explain, alone, the different experimental findings obtained by different manipulations/modulations of this current, suggesting that an additional interaction with another mechanism must be in effect, especially to explain results involving dendritic or synaptic inputs. This was originally recognized for neocortical pyramidal neurons [23], another neuronal population with a non uniform dendritic distribution of *I_h_* [24]. In this case, the best fit between model and experiments was found assuming a non uniform distribution of both *I_h_* and passive properties. Most (but not all) of the experimental findings, on the effects of *I_h_* manipulation/regulation in neocortical and hippocampal CA1 pyramidal neurons, can thus be conveniently reproduced in computational models by modifying the passive properties (i.e. the leak current). This method, however, in some cases may exaggerate the effects of *I_h_* regulation [2], [25].

Accumulating experimental evidence now demonstrates that the action of *I_h_* on synaptic integration can be more complex than previously thought [1]–[2]. In other types of neuron, a dynamic indirect interaction of *I_h_* with *K^+^* channels has been reported in principal neurons of the Medial Superior Olive [26] and in the rod photoreceptors [27], whereas a bidirectional interaction with colocalized *Na^+^−K^+^* pumps has been found to modulate excitability in mesencephalic trigeminal neurons [28]. None of these mechanisms seem to be able to take into account the experimental results discussed here, and an interaction/interplay between *I_h_* and other active or passive currents has been suggested as one of the factors that can potentially influence the role of *I_h_* in epilepsy [29]. However, this aspect has never been experimentally investigated. In this work, we showed that a shunting current that depends on *I_h_* channels can take into account virtually all experimental findings on the effects of modulators or pathological conditions that result in *I_h_* regulation. We propose that, in any given neuron, the *I_h_* may be excitatory or inhibitory according to the strength of this current. Its detailed nature, properties, distribution and, especially, the kind of interaction with *I_h_*, remain to be determined. We cannot exclude the possibility that the puzzling experimental findings discussed here are simply caused by unknown non-specific effects of the pharmacological or experimental manipulations, or non trivial (and rather arbitrary at this stage) combinations of effects from other main channels. The model results indicated the simplest solution, and more unambiguous experiments are required to characterize in more details this elusive and peculiar mechanism, which may be of fundamental importance in the development of new treatments for all those pathological and cognitive dysfunctions caused, mediated, or affected by *I_h_*. From this point of view, the model suggests several experimentally testable characteristics for *I_lk_*:

- Its reversal potential must be lower than the resting potential, and it must not inactivate with depolarization in the subthreshold range (otherwise there will be no inhibitory effect in the presence of *I_h_*, especially during dendritic stimulations);
- it must be coupled in some way to the local *I_h_* channels (to explain the experiments by George et al. [5]) and unaffected by ZD (to explain the results with LTG [4] and pilocarpine [30]);
- its dendritic distribution should follow that of *I_h_* channels (otherwise experiments showing differential effects for somatic and dendritic stimulations, such as those with LTG, cannot be explained);
- in principle, it can be a non-inactivating K^+^ current, such as that carried by TASK-like channels [31], but it is unlikely to be the *K_M_* since it does not depend on *I_h_* channels;
- it might be blocked or altered by XE991 (to take into account the consistent effect of this drug in George et al. experiments [5]).

### Conclusions for Modelers

In order to model experimental findings involving the role and effects of *I_h_*, we suggest the use of *I_lk_* as discussed in Methods, rather than adjustment of the leak current at rest. In some cases it will result in the same effect (e.g. for *lk* = 1 and *v_rev_lk_* = RMP), but the experimental findings discussed here demonstrated that this may not always be the case (i.e. *lk* and *v_rev_lk_* may be different under different cells/conditions). Also, the interaction between *I_h_* and the *I_lk_* (through the *g_h_*(*x*), see Methods) appears to be a necessary condition to correctly model any *I_h_* regulation/manipulation.

### Conclusions for Experimentalists

The results discussed in this paper suggest that any experiment studying the effects of *I_h_* should involve a careful assessment of *I_lk_* (in terms of *lk* and *v_rev_lk_*) for the specific set of cells used in the experiment. Cell-to-cell variability caused by cell specific activity-dependent changes in dendritic *I_h_* distribution [32] can shift the crossover point. Without an estimation of this effect, it will be problematic to analyze the experimental findings, especially those involving synaptic integration.

### Conclusions for the General Reader

In investigating the possible mechanisms leading to the development of new drugs that may reduce the burden related to mental disorders, it is becoming increasingly evident that targeting specific channels may confer a new level of efficacy and specificity to drug actions, with important advances for the development of ion channel based therapies. The *I_h_* is particularly involved in epilepsy, and thus any information on the intricacies of its regulation may add important clues on the possible ways to take advantage of its properties and distribution to develop new selective drugs. Here we have shown one additional factor to exploit.
